# Invasive pulmonary aspergillosis in patients with chronic obstructive pulmonary disease: a case report and review of the literature

**DOI:** 10.18632/oncotarget.16971

**Published:** 2017-04-08

**Authors:** Zhiyao Bao, Hong Chen, Min Zhou, Guochao Shi, Qingyun Li, Huanying Wan

**Affiliations:** ^1^ Department of Respiratory Medicine, Ruijin Hospital, School of Medicine, Shanghai Jiaotong University, Shanghai, China

**Keywords:** invasive pulmonary aspergillosis, COPD, Aspergillus fumigatus

## Abstract

Invasive pulmonary aspergillosis (IPA) is an infection that often occurs in immunocompromised patients and has a high mortality rate. In recent years, the reported incidence of IPA in the context of chronic obstructive pulmonary disease (COPD) has seemingly increased. The combination of factors such as long-term corticosteroid use, increasing rate of bacterial exacerbations over time, lung immune imbalance, and malnutrition are responsible for the emergence of IPA in COPD patients. A diagnosis of IPA in COPD patients is difficult to make, which explains the delay in antifungal therapy and the high mortality rate. The purpose of this study is to increase the recognition and improve the outcomes associated with this situation through the description of our case. In patients in which IPA is suspected, comprehensive analysis of their clinical manifestations, imaging, microbiology and serological examination results are effective means of increasing the rate of reliable diagnosis. If the patient’s condition permits, a pathological specimen should be obtained as soon as possible.

## INTRODUCTION

*Aspergillus fumigatus* is a saprophytic fungus that is capable of causing a wide range of conditions, including allergic bronchopulmonary aspergillosis (ABPA), aspergillomas and invasive aspergillosis (IA). Among them, IA is the most common and the most severe. Additionally, this opportunistic disease occurs predominantly in immunocompromised hosts such as those with hematologic malignancy, allogenic bone marrow transplantation, solid organ transplantation and late-stage HIV infection, etc. [1]. Aside from these high-risk groups, many cases of IA have been reported in nontraditional hosts, especially patients with chronic obstructive pulmonary disease (COPD) [2]. In this article, we will describe the clinical characteristics of a patient with COPD combined with invasive pulmonary aspergillosis (IPA) who were diagnosed by fiberoptic bronchoscopy in our hospital to improve our understanding of this disease.

## RESULTS

An 83-year-old woman with a history of exposure to biofuels and stage IV COPD in group D who was under treatment with inhaled corticosteroids (250μg of fluticasone twice daily for more than 6 years) and intravenous systemic methylprednisolone (40-80 mg per day intermittently) was hospitalized due to the exacerbation of dyspnea and fever. She also had a history of hypertension, diabetes mellitus and cardiac insufficiency.

**Figure 1:**
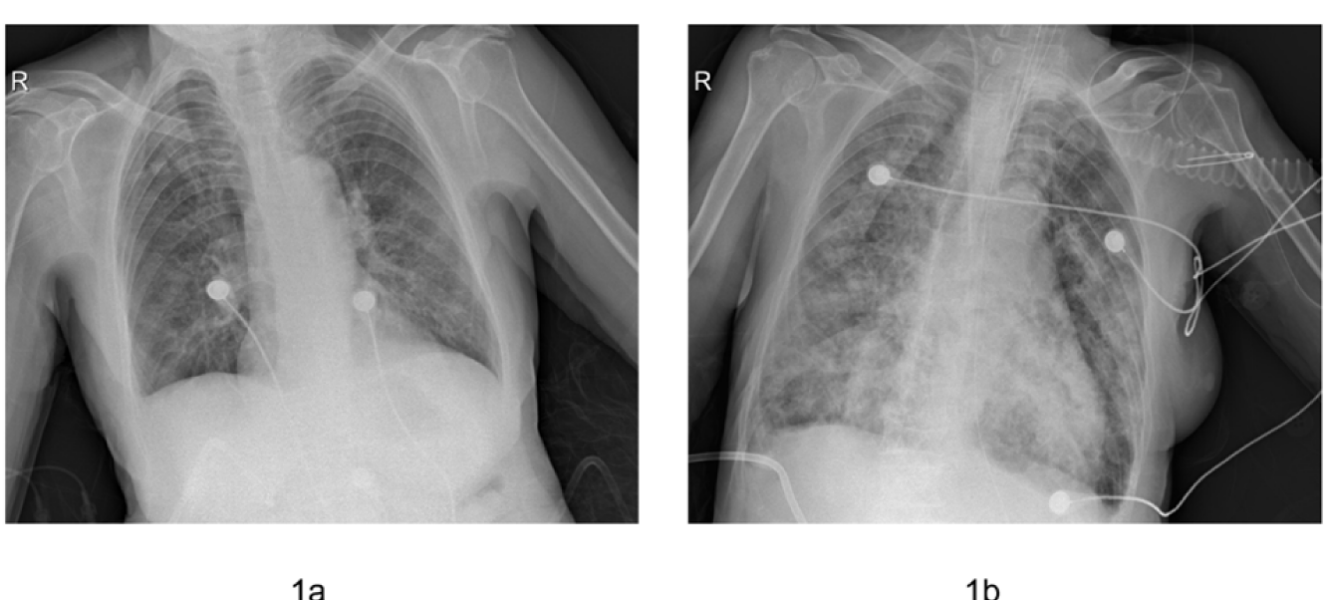
Comparison of the chest X-ray performed upon admission to the hospital (a) and upon intubation (b)

At admission, chest X-ray showed opacity in both lower lobes (Figure 1a). The blood cultures and serum antibody tests for chlamydia and mycoplasma were negative. Treatment was started with cefminox and levofloxacin, high doses of a systemic corticosteroid (methylprednisolone 40 mg iv twice a day) and non-invasive ventilator support.

Three days later, the patient's condition continued to deteriorate. Therefore, she was intubated and given mechanical ventilation. Chest X-ray showed an enlargement of the opacity in all the lobes (Figure 1b). Additionally, her procalcitonin (PCT) levels increased from 0.12 ng/ml (at the start of antibiotic therapy) to 1.04 ng/ml. Consequently, the empirical antibiotic therapy was changed to linezolid and faropenem. Given that direct examination of the patient's sputum revealed elements that were compatible with septate hyphae, the β-1.3-glucan test of her serum was positive (>1000 ng/L). In addition, as her clinical symptoms did not improve, intravenous voriconazole was started immediately (400 mg kg^-1^ day^-1^).

**Figure 2:**
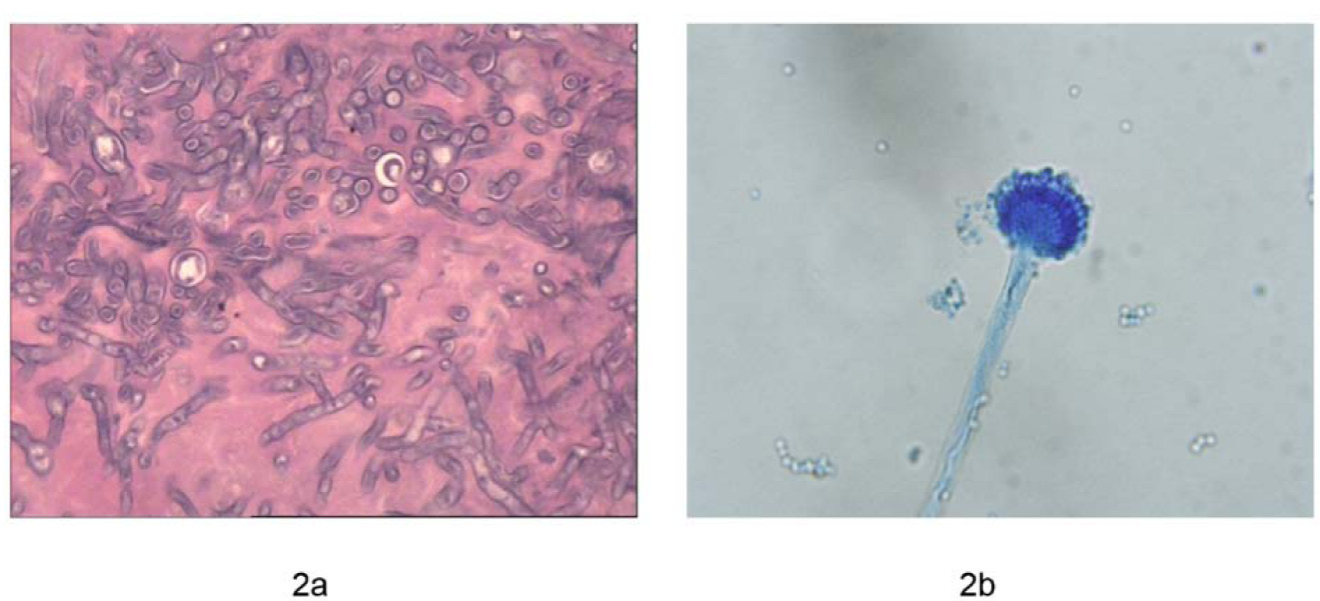
Morphology under the microscope **a**. Pathology shows the dichotomously branched and septate hyphae, HE 10×40. **b**. The typical conidial head of *A. fumigatus* in lactophenol cotton blue stain, 10×40.

**Figure 3:**
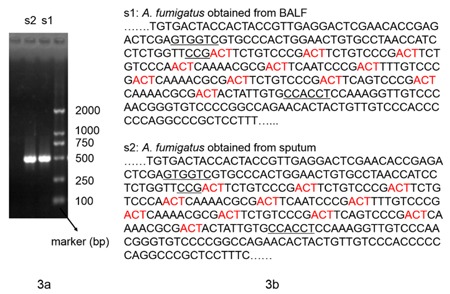
Molecular biology analysis of isolated *A. fumigatus* strains **a**. The electrophoresis results of the β-tubulin region. **b**. The partial sequencing results of the CSP gene.

Furthermore, bedside fiberoptic bronchoscopy was performed to confirm the pathogenic result. It demonstrated that the airway was edematous and covered with sticky purulent secretion. Furthermore, there was an excrescence at the left lower lobar bronchus. Dichotomously branched and septate hyphae were found in the tissue sections of the excrescence (Figure 2a). *Aspergillus fumigatus* was also isolated from both the sputum and the bronchoalveolar lavage fluid (BALF), which showed the typical conidial head of *A. fumigatus* in lactophenol cotton blue stain (Figure 2b). Moreover, it was identified by the sequencing of the β-tubulin region and was genotyped by CSP, a highly discriminatory and reproducible procedure (Figure 3). Both isolates had the same genotype t02. In addition, the antifungal susceptibility of the isolates was determined using the CLSI M38-A2 procedure. They were found to be susceptible to common antifungal drugs. The MICs for voriconazole, itraconazole, posaconazole and amphotericin B were 0.25, 0.25, 0.03 and 0.5μg/ml, respectively.

Although the patient received antifungal therapy as early as possible, she died 10 days after admission.

## DISCUSSION

Although prolonged neutropenia and immunosuppression in association with solid organ transplantation have been identified as the most common underlying conditions in patients with IPA. It is reported that there are 200,000 estimated annual cases of IA, approximately 50% of which occur in patients with hematological malignancy. Additionally, the incidence rate of IA in ICU patients ranges from 6.1 to 57 out of 1000 ICU admissions [3]. However, patients with other underlying conditions, especially COPD, are receiving increasingly more attention. Such patients might have increased susceptibility to invasive fungal infection for several reasons: (a) the common use of long-term or repeated short-term steroids, (b) frequent hospitalization and broad-spectrum antibiotic treatment, leading to selective pressure, which favors fungal pathogens, (c) structural changes in the lung architecture related to the pulmonary condition, and (d) comorbid conditions such as diabetes mellitus or malnutrition [4].

Diagnosis of IPA in COPD patients is difficult. As underlying disease can mask the findings of *Aspergillus* lung invasion, the classic symptoms of IPA such as fever, cough, chest pain and hemoptysis are less common in COPD patients than in patients with blood diseases [5]. Furthermore, the classic radiological findings of IPA such as the halo sign or the air crescent are less common in patients with COPD than in neutropenic patients [6]. The most frequent radiological finding is infiltration [7] that is non-specific and might correspond to the exacerbation of COPD that is caused by bacterial pulmonary infection, which is frequently encountered especially in chest disease clinics. The main clinical characteristic, as illustrated by our patient, was the exacerbation of dyspnea, as evidenced by the non-specific antibiotic-resistant pneumonia with an enlargement of infiltration in the lower lobes in her chest X-ray.

Microbiological confirmation of IPA in COPD patients is limited. The positive predictive value of the isolation of *Aspergillus* in lower respiratory samples is low due to the high proportion of patients who are colonized by *Aspergillus* spp. However, increasingly more studies have reported that the isolation of *Aspergillus* spp. from the lower respiratory tract of COPD patients should not be routinely dismissed as colonization, especially in those with whom antibiotics and steroids have poor efficacy. Furthermore, they should be carefully evaluated to exclude IPA [8]. There is no consensus regarding how to evaluate these patients further, but the following recommendations are offered: closer observation and additional studies to include features such as high-resolution CT (HRCT) of the chest, serological studies to detect Aspergillus antigens, possible bronchoscopy and empirically driven antifungal therapy with critically ill patients. In our patient, *A. fumigatus* was isolated from both the sputum and the BALF, yielding the same genotype. Given this finding combined with the patient's clinical features, we employed bronchoscopy to obtain the histological confirmation.

Extensive studies have been conducted to assess the value of bronchoscopy and BALF in the diagnosis of IPA. Whereas these studies primarily involved immunocompromised patients, they did not include COPD patients. There are limited reports on the role of transbronchial biopsies in the diagnosis of IPA in COPD patients, which shows that this intervention did not add much to the yield but increased the risk of complications. In a retrospective analysis conducted by Samarakoon et al., transbronchial biopsy was performed in six of sixty-five COPD patients with IPA and was positive for invasion of the lung parenchyma in all six of them [6]. It appears that transbronchial biopsies might be a useful test for use with COPD patients with whom there is a high clinical or radiological suspicion of IPA or who do not respond to conventional antibiotics. Transbronchial biopsy was performed with our patient due to the establishment of an artificial airway. Based on the isolation of *Aspergillus fumigatus* from the BALF and histological confirmation, our patient met the criteria for proven IPA (Table 1) [9].

**Table 1:** The diagnostic criteria for IPA in COPD patients

Proven IPA	Histopathological or cytopathological examination of a needle aspiration or biopsy specimen obtained from any pulmonary lesion that has been present for <3 months showing hyphae that are consistent with Aspergillus and evidence of associated tissue damage if accompanied by any one of the following: 1) positive culture of Aspergillus spp. from any lower respiratory tract (LRT) sample, 2) positive serum antibody/antigen test for A. fumigatus (including precipitins), or 3) confirmation that the observed hyphae are those of Aspergillus by a direct molecular or immunological method and/or culture.
Probable IPA	The same as for proven IPA but without confirmation that Aspergillus is responsible (points 1, 2 and 3 are not present or tested) OR COPD patient who is usually treated with steroids and severe according to GOLD (stage III or IV) with recent exacerbation of dyspnea, suggestive chest imaging (radiograph or CT scan; <3 months) and one of the following: 1) positive culture and/or microscopy for Aspergillus from LRT, 2) positive serum antibody test for A. fumigatus (including precipitins), or 3) two consecutive positive serum galactomannan tests.
Possible IPA	COPD patient who is usually treated with steroids and severe according to GOLD (stage III or IV) with recent exacerbation of dyspnea, suggestive chest imaging (radiograph or CT scan; <3 months) but without a positive Aspergillus culture or microscopy from LRT or serology.
Colonization	COPD patient with a positive Aspergillus culture from LRT without exacerbation of dyspnea, bronchospasm or new pulmonary infiltrate.

Even though amphotericin B has been used as the first-line antifungal agent in the treatment of IPA for several years, voriconazole is currently approved as the initial treatment for invasive aspergillosis in many patients with IPA [1]. Voriconazole is available in both intravenous and oral formulations and has a milder side effect profile. Additionally, it is tolerated to a greater extent than amphotericin B [10]. Posaconazole and echinocandin derivatives such as caspofungin, micafungin and anidulafungin are other agents that can be used in the treatment of IPA refractory to standard treatment or if the patient cannot tolerate first-line agents [11]. Although our patient received voriconazole therapy and the *Aspergillus fumigatus* that was isolated from her lower respiratory tract specimen was sensitive to it, she still died within a short amount of time. We speculate that her rapid death was primarily related to the delay in the diagnosis of IPA, as it is not routinely considered in this patient population. Other potential factors that were associated with her death included her older age, poor pulmonary reserve and multiple comorbid illnesses.

Our study has a few limitations. First, we only detected the patient's serum level of β-1.3-glucan, which is a cell wall component of not only filamentous fungi but also yeasts. Hence, its specificity is limited. However, we did not detect galactomannan (GM) in our patient's bodily fluids due to the lack of the testing reagent. GM is a polysaccharide cell wall component that is released by *Aspergillus* during its hyphal growth phase. Originally, it was confirmed that the GM test was a useful method to diagnose IPA in immunocompromised patients. Currently, increasingly more studies have indicated that the test is also a sensitive and specific way to diagnose IPA in patients with COPD, especially in the BALF [12,13]. Moreover, it has been shown that a positive GM test in COPD patients with IPA development who are in intensive care might be significant in terms of mortality [14]. Hence, the GM test is a highly recommended method to diagnose IPA in patients in which the condition is suspected, especially those whose pathological evidence cannot be obtained. Second, although isolated *A. fumigatus* is sensitive to triazoles, it can be highly virulent, causing the unfavorable prognosis. Further studies of virulence in these isolates such as the determination of the elastase activity index could be of interest.

In conclusion, the mortality rate in association with IPA in COPD patients is high because of the difficulty surrounding its diagnosis. Hence, comprehensive analysis of COPD patients’ clinical manifestations, imaging, microbiology and serological examination results are effective means of increasing the rate of reliable diagnosis. If the patient's condition permits, a pathological specimen should be obtained as soon as possible. This is a recommendation for general alertness of the episodes, particularly in patients with COPD who receive prolonged therapy with a steroid, antibiotics, etc.

## MATERIALS AND METHODS

### The diagnosis and treatment of a patient with COPD and IPA were reviewed in our hospital

#### Isolates

Two isolates were investigated in this study. One was from the sputum, whereas the other was from the bronchoalveolar lavage fluid (BALF). They were identified as *A. fumigatus* based on their macroscopic and microscopic characteristics and their ability to grow at 48°C.

#### DNA isolation

The genomic DNA was isolated from the conidia harvested from cultures grown at 30°C for 3-4 days. DNA extraction was performed, as previously described [15]. The DNA yield and purity were estimated using UV absorbance measurements.

#### Identification of the isolates

Both the isolates were identified by the sequencing of the β-tubulin region [16]. The amplification reactions of 50μl contained 2μl (concentration was 10μM) of each amplification primer (5’-AATTGGTGCCGCTTTCTGG-3’ and 5’-AGTTGTCGGGACGGAATAG-3’), 4μl dNTPs, 0.25μl of Taq DNA polymerase (TaKaRa) and 2μl of DNA in 1×reaction buffer. The cycling program consisted of a 5-min activation/denaturation step at 95°C, followed by 35 cycles of 30 s at 94°C, 45 s at 55°C and 1 min at 72°C. Furthermore, additional incubation for 4 min at 72°C was included before the reactions were cooled to room temperature. The products were visualized in 1.5% agarose gel.

#### CSP typing

Part of the CSP gene was amplified using the following primers: 5’-TTGGGTGGCATTGTGCCAA-3’ and 5’-GGAGGAACAGTGCTGTTGGTGA-3’. In addition, the reaction system was the same as above. CSP sequence types were assigned to isolates as defined in the proposed nomenclature [17].

#### Antifungal susceptibility test

Two isolates were examined regarding their susceptibility to voriconazole, itraconazole, posaconazole and amphotericin B in a broth microdilution susceptibility test [18].
